# Conceptualizations of Addiction and Moral Responsibility

**DOI:** 10.3389/fpsyg.2019.01483

**Published:** 2019-06-28

**Authors:** Jostein Rise, Torleif Halkjelsvik

**Affiliations:** Department of Alcohol, Tobacco and Drugs, Norwegian Institute of Public Health, Oslo, Norway

**Keywords:** addiction, agency, free will, moral judgment, conceptualizations, responsibility

## Abstract

The present study explored the connection between conceptualizations of addiction and lay people’s inferences about moral responsibility. In Study 1, we investigated how natural variations in people’s views of addiction were related to judgments of responsibility in a nationwide sample of Norwegian adults. In Study 2, respondents recruited from Mechanical Turk were asked to consider different conceptualizations of addiction and report on how these would affect their judgments of moral responsibility. In Study 3, we tested whether manipulating conceptualizations through textual information and through the framing of addiction in terms of states versus behavior could influence participants’ judgments of moral responsibility. We found that attributions of moral responsibility were lower when addiction was connected to diseases and disorders, such as dysfunctional processes in the brain, and greater when addiction was associated with agency and addictive behaviors. In conclusion, different conceptualizations of addiction imply different moral judgments, and conceptualizations are malleable.

Addiction as a phenomenon is a puzzle, paradox, and slippery concept for which definitions and classifications in diagnostic systems have changed with cultural, political, and scientific developments (Berridge et al., 2014; Room et al., 2015). A key concept in the discourse about addiction is the question about moral responsibility (see e.g., Morse, 2004; Foddy, 2011; Levy, 2011; Uusitalo, 2011). Are individuals addicted to substances morally responsible for their use, or does addiction represent a form of involuntary behavior? The starting point of the present study is that the answer to this question depends on the way addiction is conceptualized, that is, how people view and describe addictions. Knowledge of the connection between judgment of moral responsibility and conceptualizations of addiction may be important for understanding, and potentially changing, how addicted individuals are treated in society. Moral judgments may have a range of consequences from how drug policies are formed to how professionals in the healthcare and criminal justice systems behave toward addicted persons (cf. Pickard and Pierce, 2013). The concept of moral responsibility has been treated in diverse bodies of literatures, from which three lines of inquiry may be particularly relevant: scientific addiction models, research on stigma and attribution, and the contemporary literature on free will and agency.

## Addiction Models

The scientific discourse about addiction has been dominated by two models: the disease model and the choice model (Morse, 2004; Henden et al., 2013; Uusitalo et al., 2013). The former considers addiction as following a disease-like course, with behaviors that have taken control of the person–so-called compulsive actions. A modern version of the disease model is the view of addiction as a brain disease (see e.g., Kennett and McConnell, 2013). The brain disease model holds that neural processes and chemical reactions following repeated intake of drugs cause lasting brain changes so that the reward system is hijacked and governs the motivations behind addictive behaviors. This model has recently been challenged from a number of perspectives (see Heyman, 2009; Henden et al., 2013; Lewis, 2015; Heather et al., 2017; Pickard, 2017a). In contrast to the brain disease model, the choice model holds that addictive behaviors are governed by universal principles of choice and motivation. The choice model has been referred to as the successor of the moral model of addition (Kennett and McConnell, 2013), where addiction was considered a moral failure and addicts could be perceived as people of bad character (see Pickard, 2017b). However, moral considerations are not core features of modern choice theories of addiction (cf. Heyman, 2009).

Recently, several authors have argued in favor of views that place addiction somewhere in the middle of a continuum between nonvoluntary behavior and voluntary actions (Henden et al., 2013; Holton and Berridge, 2013; Heather, 2017a). This middle ground involves *excusing conditions* for addictive behaviors, meaning that there are strong forces at play that are difficult, but not impossible, to resist (see Morse, 2004; Levy, 2011; Pickard and Pierce, 2013).

## Stigma and Attribution

Individuals addicted to drugs are heavily stigmatized and viewed by lay people as more dangerous and blameworthy than individuals with mental illness or physical disabilities (Corrigan et al., 2009). Several factors appear to moderate the level of stigmatization (e.g., Corrigan et al., 2001, 2002; Pinfold et al., 2003; Schulze et al., 2003). For instance, in a recent survey, stigmatization of people with drug addiction was influenced by factors related to the stigmatized person (such as gender, age, and duration of addiction) and demographic characteristics of the person making the judgment (Sattler et al., 2017). The authors of the study found their results to be fairly consistent with Weiner’s attribution theory (e.g., Weiner, 1995, 2006). A core assumption in the attribution theory is that controllability of a stigmatized behavior is consequential for perceived responsibility, which, in turn, is consequential for social emotions and outcomes such as helping behavior. Thus, perception of responsibility plays a central role in a process linking inferences regarding causes and controllability to emotional and behavioral consequences (Weiner, 1995; see also Shaver, 1985).

## Free Will

Lay people seem to associate addiction with a loss of free will (Vonasch et al., 2017). Because free will is held to be a prerequisite for an agent to be punished for wrongdoing and praised for doing well, a number of scholars have posited a close relation between free will and moral responsibility (see Nahmias, 2018). The main debate in philosophy revolves around whether free will and moral responsibility are compatible with determinism–the idea that whatever happens is fully determined (caused) by previous events and the laws of nature (Mele, 2006). While *compatibilists* hold that free will and moral responsibility are compatible with determinism, *incompatibilists* deem that if determinism is true, then humans cannot have free will and be morally responsible for their actions. Results regarding this issue from empirical research on lay intuitions are divided (Cova and Kitano, 2014). While Nahmias and Murray (2010) claim that ordinary people are natural compatibilists, Nichols and Knobe (2007) claim that they are natural incompatibilists.

A recent psychological model of free will does not focus on whether or not lay people believe in free will but on what they mean by free will (Monroe and Malle, 2010, 2015). In essence, free will means that choices are unconstrained by internal and external circumstances (Monroe and Malle, 2010, 2015; Feldman et al., 2014; Vonasch et al., 2018). In one study, Monroe et al. (2016) found that after accounting for perceived choice capacities, nothing was left for a general and abstract belief in free will to account for lay peoples’ judgment of an agent’s immoral behavior. This suggested that a general belief in free will is a shorthand lay people use for the ascription of these capacities.

Thus, lay people’s ascription of moral responsibility associated with addiction can be placed on a continuum from low to high (cf. Sinnott-Armstrong, 2013), and underlying this continuum is a model of freedom of action and free will as capacities to make decisions and exercise control. By this account, one should not treat free will and freedom of action as all-or-nothing properties (Nahmias, 2018). Of particular interest for the present research are the results from experimental philosophy studies on free will and determinism demonstrating that lay people’s responses to questions of moral responsibility can vary dramatically depending on the way researchers formulate the scenario (see Cova and Kitano, 2014 for a review).

## Conceptualizations of Addiction and Moral Responsibility

As part of the 2012 Queensland Social Survey in Australia, Meurk et al. (2014) found that considering addiction as a brain disease or as an ordinary disease did not affect beliefs about stigma nor belief about the use of coerced treatment on and imprisonment of heroin users. Furthermore, the respondents’ views on causes of addiction were inconsistent predictors of these beliefs. Meurk et al. (2014) argued that these results corroborated those of their prior qualitative studies, indicating that new information about addiction, in particular information portraying addiction as a brain disease, would not produce dramatic shifts in people’s beliefs about addiction. Similarly, Rather (1991) investigated lay models of alcohol addiction and reported no effect of the manipulation of a disease model versus social-learning model of alcohol addiction on attitudes toward alcoholics or judgments of deservingness of help, even though the manipulation affected beliefs about the causes of addiction.

The above studies did not directly concern moral judgments but hinted at the difficulties in linking conceptualizations of addiction to attributions of moral responsibility among lay people, at least in terms of changing such conceptualizations. In a recent and highly relevant study, Racine et al. (2017) compared the effect of three types of neuroscientific information about addiction (alcohol and cocaine) on the attribution of free will: (1) a textual neuroscience description of addiction, (2) neuroimages of a nonaddict’s and of an addict’s brain, (3) a combination of text and neuroimages, and (4) a control condition with no information. A factor analysis of a scale measuring lay beliefs about whether addicts have free will revealed two distinct free will factors denoted Responsibility and Volition. One hypothesis was that a neuroscience perspective of addiction would reduce the attribution of free will and subsequently the blame. However, they only found a significant effect of the combined image and textual description on the volition subscale in terms of diminished free will for cocaine addiction. Racine et al. (2017) argued that the results indicate that naturally occurring neuroscientific information about addiction might have limited effects on attributions of free will (responsibility and volition), and, accordingly, that the merits of the brain disease model may have been overstated.

The above studies involved efforts to change conceptualizations of addiction. Another question in the literature of addiction is whether, and how, natural variations in the lay peoples’ conceptualizations matter for moral judgments. Research on perceptions of addictions to different types of behavior suggests that the type of addiction (i.e., type of substance) is consequential for moral judgments. In a nationwide study among Swedish adults, Blomqvist (2009) explored responsibility judgments for nine different types of addiction. He distinguished between responsibility for the onset of a problem and responsibility for solving the problem (see Brickman et al., 1982). Addiction to tobacco fit into a *moral model*, where lay people perceive users as responsible for both the onset and the solution to the problem. Addiction to alcohol, sedatives, and cannabis were placed within the *compensatory model*, where users are responsible for the solution of the problem but not the onset. Hard drug addicts fit into a combination of the *medical model* (neither responsible for the onset nor for its solution, i.e., they have a disease and should receive treatment) and the *enlightenment model* (responsible for the problem but not for the solution of the problem), implying that addicts are victims that need external help to overcome the addiction. The study by Blomqvist (2009) and similar studies (Halkjelsvik and Rise, 2014; Rise et al., 2014) suggest that conceptualizations of addiction, in particular those connected to beliefs about the causes of addiction (see also Weiner et al., 1988), can be consequential for moral judgments.

## The Present Research

The issue of how conceptualizations of addiction are linked to moral responsibility can be approached in several ways. When a person holds a certain view of addiction, what does this entail in terms of moral judgments? When a person receives and accepts a certain description of addiction, what does he/she believe this implies in terms of moral responsibility? Can information or the framing of addiction shape people’s own beliefs regarding moral responsibility? These are different questions, but they all pertain to the relation between conceptualizations of addiction and moral judgments. When one describes addiction in research and in the media, knowledge about what the different labels and descriptions imply in terms of moral judgments can be valuable, particularly if the words have an impact on other people’s moral judgments.

We explored the connections between conceptualizations of addiction and moral judgments in three studies, using different approaches. In Study 1, we recruited a broad sample of the Norwegian population and used a wide array of textual descriptions reflecting the ways addiction has been described in the literature. We investigated how variations in people’s endorsement of these descriptions related to their judgments of responsibility. In Study 2, we explicitly asked people to accept different conceptualizations and then investigated how this would affect judgments of moral responsibility. In Study 3, we tested whether we were able to manipulate judgments of responsibility through textual information about addiction and through changing the object of evaluation by framing addiction in terms of addictive states versus addictive behaviors.

### Study 1

People have different backgrounds, values, and ideologies, and it is reasonable to assume that there is substantial variation in people’s views of addiction. The same individual can have different views of addiction, depending on the type of addictive behavior involved (e.g., cigarette smoking versus use of heroin). In Study 1, we attempted to exploit this natural variation in conceptualizations of addictions by exploring lay people’s ratings of a range of addiction descriptors that were derived from the scientific literature on addiction.

Each respondent rated several types of addiction which enabled us to explore two types of effects in Study 1, one based on between-person differences in conceptualizations of addiction and another based on within-person differences. Both effects may be informative regarding the relation between conceptualizations and moral judgments; however, only the latter removes time-invariant confounding. We controlled for the overall differences between types of addiction (i.e., the averages across the sample of individuals), as these may be heavily influenced by the legal status and the prevalence of the addictive behavior.

#### Methods

##### Data and Sample

The recruitment panel of an independent research company was used to invite a representative sample of Norwegians aged 20–70 with access to the internet (i.e., the online population). Of the 2,964 invited to participate, 2,037 responded to at least one question in a large survey on addictions and related issues. Except for one analysis (*N* = 1,853), the number of respondents ranged from 1979 to 2011 in the statistical analyses. The mean age of the sample was 47, SD = 14; 50% were women. Results from the same survey have previously been reported in Melberg et al. (2013), Rise et al. (2014, 2015), but none of the present analyses have been published before. None of the studies reported in the present article required ethics approval per our institution’s guidelines and Norwegian law. We did not collect IP addresses or any personal or sensitive information. Participation was voluntary; participants were informed that their responses would be used in research; and they were asked to consent by proceeding to the survey questions.

#### Measures

##### Types of Addiction

The study involved ratings of addiction to cocaine, hashish (cannabis), alcohol, gambling, smoking, amphetamine, sedatives, snus (Swedish moist snuff)], and heroin. Participants rated all nine addiction types in terms of 13 different addiction descriptors.

##### Addiction Descriptors

After the initial text: “Addiction to [type of addiction] is/represents…”, respondents rated their level of agreement with 13 different descriptors of addiction (see Table 1) on a seven-point scale from “Fully disagree” (coded 1) to “Fully agree” (coded 7). As an example, the participants rated the level of agreement with the statement “Addiction to Cocaine is/represents…reduced willpower”. The descriptors were based on an informal survey of the literature and reflected conceptualizations by lay people and scientists (see Rise et al., 2015).

**Table 1:** Unstandardized regression coefficients from 13 regression analyses predicting responsibility judgments from endorsement of addiction descriptions, sorted from negative to positive on the within-subject effects, Study 1.

	Within	SE	Between	SE	*N*	Obs.
Ordinary disease	−0.07^*^	0.010	−0.12^*^	0.019	1993	16,478
Mental disorder	−0.05^*^	0.008	−0.04	0.017	1979	15,855
Conflict between strong desires	−0.02	0.009	0.06^*^	0.020	1933	14,813
Compulsive behavior	−0.00	0.008	0.10^*^	0.021	1994	15,985
Reduced self-determination	0.00	0.008	0.12^*^	0.020	1996	16,347
Strong urge	0.01	0.010	0.10^*^	0.027	2008	16,586
Strong appetite	0.01	0.009	0.03	0.015	1853	13,444
Craving	0.02	0.010	0.12^*^	0.030	2011	16,595
Obsession	0.02	0.009	0.14^*^	0.023	2004	16,528
Reduced rationality	0.02^*^	0.008	0.23^*^	0.023	2008	16,601
Reduced willpower	0.04^*^	0.009	0.20^*^	0.022	2000	16,355
Reduced morality	0.05^*^	0.008	0.18^*^	0.019	1989	16,167
Habit	0.06^*^	0.009	0.10^*^	0.022	2008	16,439

Obs = number of observations. Control variables in the regressions: addiction type (nine categories) and questionnaire version.

* p < 0.01.

##### Responsibility Judgments

The outcome measure was ratings of whether a person addicted to (type of addiction) should be held responsible for becoming addicted to the substance/behavior. The response scale was from “To a very small extent” (coded 1) to “To a very large extent” (coded 7). The option “do not know” was coded as missing.

##### Statistical Analyses

Analyses were performed in STATA 14.1 using the “mixed” command with maximum likelihood estimation and robust standard errors; *p*’s were based on the default large-sample tests. We ran separate regression models for each of the 13 addiction descriptors in Table 1. In each analysis, the outcome measure was a variable comprising the responsibility ratings for all the nine types of addiction. Type of addiction was controlled for by dummy indicators, and the predictor of interest was the endorsement of the given descriptor (i.e., the extent to which respondents agreed that a descriptor is/represents a given addiction). We included two different terms in the regressions to estimate the effect of endorsement of a given descriptor on moral judgments. One term represented the between-individuals effect and was estimated by a variable consisting of each individual’s mean endorsement of the given descriptor across addiction types; another term represented the within-individual effect and was estimated by the endorsement ratings minus the respective individual’s mean endorsement (for more on this “within-between” approach, see Bell and Jones, 2015; Snijders and Bosker, 2015, p. 58). For example, if the regression coefficient of the within-effect for the item “Conflict between strong desires” is −0.02, it means that a within-person difference of one unit in ratings of the level of agreement with the statements “Addiction to [addiction type] is/represents a conflict between strong desires” gives a 0.02 unit decrease in the ratings of responsibility. This effect is based on the variation within the individuals, in their ratings of the nine different addiction types for the conflict-between-desires items, after controlling for mean ratings of the nine addiction types. If the regression coefficient of the between-effect is 0.06, it means that a participant with an average level of agreement of 5.5 on the items concerning “Conflict between strong desires” typically rate judgments of responsibility 0.06 points higher than a participant with an average rating of 4.5. Thus, the between-participant effect can be positive even if the within-participant effect is negative. The within-effect can be considered as similar to the type of coefficient one would obtain with so-called fixed-effect (FE) models used by economists (see Bell and Jones, 2015), and the between-effect approximately represents the effect one would obtain if we for each participant aggregated his/her nine ratings of agreement with a given statement and used this aggregated score as a predictor of the participants’ average level of responsibility judgments. In addition to the above within-individual, between-individual, and addiction type variables, the regressions included subjects (ID variable for each respondent) as random intercepts and questionnaire version^1^ as a dummy-coded, fixed-effect control variable. We used a threshold of *p* < 0.01 to identify the most promising effects in Study 1.

#### Results and Discussion

Table 1 presents the results of the 13 separate analyses of the addiction descriptors, ranked by the strength and direction of association with the responsibility measure. If we focus on the within-subject effects, as these adjust for time-invariant confounders (such as a general tendency to agree/disagree with survey questions, or the main effects of respondents’ backgrounds), we found that endorsement of the descriptors “reduced rationality”, “reduced willpower”, “reduced moral competence”, and “habit” were all associated with a higher level of responsibility ratings, while “ordinary disease” and “mental disorder” were negatively related to responsibility ratings.

Thus, we identified several descriptors that were associated with responsibility, notably those referring to disease or disorder, and those related to reduced ability to make the right decisions (reduced rationality, reduced morality) or control impulses (reduced willpower and habit). The descriptors conceptualizing addition as strong motivation (urges, appetites, cravings, and obsessions) were not related to judgments of responsibility in terms of within-person effects. However, we found generally stronger associations between judgments of responsibility and endorsements between individuals than within individuals. We do not have a definite explanation for this, but it might be, for example, that people’s general conceptualizations of addiction matter more for responsibility judgments than do perceived differences between addiction types, or that the larger between-person effects simply reflect omitted variables related to participants’ characteristics.

### Study 2

Although we believed that the results from Study 1 hinted at a causal link from addiction conceptualizations to responsibility judgments, other reasons might explain the covariation. In Study 2, we wished to directly probe whether different descriptions implied different responsibility judgments by asking respondents to accept different conceptualizations and then judge the moral responsibility for addiction. For this purpose, we selected the most promising addiction descriptors from Study 1, that is, the descriptors that appeared to have within-person effects. The within-person effects are not confounded by stable characteristics of the participants (e.g., if younger participant were less familiar with the term “habit” and also more lenient in terms of ratings of responsibility, this would give a positive correlation between the two). In addition, we included two other descriptors that were not among the items in the large survey used in Study 1. The descriptor “brain disease” was included because it has become commonplace to define addiction as a chronic, relapsing brain disease (e.g., Leshner, 1997), and the same is the case for the label “irresistible desire” (Morse, 2004; Foddy, 2011).

The purpose of Study 2 was to identify conceptualizations of addiction that entailed higher or lower attributions of responsibility. Instead of exploiting existing natural variations, we asked about moral responsibility under different conceptualizations of addiction. We adjusted the wording of the responsibility question to underline the *moral* dimension of responsibility, and instead of responsibility for *becoming* addicted, we asked about the moral responsibility of *being* addicted, as these may differ (see e.g., Weiner et al., 1988).

#### Method

Forty-five respondents living in the United States were recruited from Amazon Mechanical Turk (Mturk). We did not collect any demographic information (but see e.g., Difallah et al., 2018, for typical characteristics of Mturk respondents).

In this within-subject study, the questions had the following format: “Given that drug addiction is [descriptor], to what extent are addicts morally responsible for their addiction?” Participants were asked to make judgments of moral responsibility for eight descriptors. The descriptors are presented in Table 2. Moral responsibility was measured on a scale from “not responsible at all” (0) to “fully responsible” (5). We did not specify the type of drug addiction. After responding to the eight descriptors, participants completed another set of survey items. The results of these are not reported here but were used in power calculations for Study 3.

**Table 2:** Mean ratings of moral responsibility for eight descriptors, sorted from low to high, Study 2.

Given that drug addiction is…	*M* (SD)
…a mental disorder	2.51 (1.44)
…a brain disease	2.76 (1.56)
…an ordinary disease	2.93 (1.52)
…an irresistible desire	2.98 (1.56)
…a form of reduced rationality	3.00 (1.40)
…a form of reduced moral competence	3.29 (1.44)
…a strong habit	3.44 (1.23)
…a form of reduced willpower	3.58 (1.14)

#### Results and Discussion

Table 2 shows the mean levels of moral responsibility ratings for the various descriptive labels of addiction, sorted from low to high levels of responsibility. The lowest moral responsibility ratings were made when addiction was defined as a disease or a disorder, and the highest moral responsibility ratings were made when descriptions directed attention toward reduced moral and rational capacities, and reduced willingness to control impulses. The moral responsibility rating of addiction as an irresistible desire was in the middle, which resulted in a pattern very similar to the ordering from diseases, *via* motivations, to reduced capacities in Study 1. A repeated-measures ANOVA obtained a *p* <0.0001, *F* (4.4, 185.1) = 8.22, Eta squared = 0.16, for the test of any differences between the ratings. The results suggest that if one succeeds in changing the way addiction is represented, this could potentially influence judgments of moral responsibility.

As in Study 1, the differences in ratings appeared to reflect a continuum from uncontrollable states to reduced capacity to choose, which is consistent with the ideas from the literature on addiction models, attribution theory, and the psychological model of free will, as presented in the introductory sections. However, it is noteworthy that the mean ratings fell within a rather narrow interval at the higher end of the scale (2.5–3.6 on a scale from 0 to 5, all medians and modes were either 3 or 4). This suggests that lay perceptions may have more in common with recent models of addiction (Henden et al., 2013; Holton and Berridge, 2013; Heather, 2017a) than with a pure choice model or a pure disease model.

### Study 3

In Study 3, we extended the conceptualization of addiction beyond the use of simple addiction labels by providing more detailed information about processes underlying addiction. Although the label “mental disorder” received the lowest ratings of moral responsibility in the previous studies, we believed that the brain disease conceptualization would be more relevant in terms of contemporary debates, and perhaps also easier to alter through provision of information. Scientists are increasingly discovering more about the neural mechanisms underlying addiction, and accumulated evidence suggests that repeated drug use leads to long-lasting changes in the brain. According to the brain disease model of addiction, these changes result in hijacking of the brain’s reward system, impairing the autonomy and restricting addicted persons’ ability to abstain from drugs, frequently denoted compulsive use (Henden et al., 2013; Pickard, 2017a,b). The modern lay person will be increasingly exposed to such reductive, mechanistic behavioral explanations couched in the neuroscientific language of neural and chemical processes. The slogan “my brain made me do it” has already become a salient feature of media, and people tend to ascribe free will and moral responsibility only to agents whose actions can be understood in terms of mental states (i.e., beliefs, desires, and intentions; Nahmias et al., 2007; De Brigard et al., 2009). Accordingly, we exposed one group of participants to detailed descriptions of brain mechanisms related to repeated drug use to see whether this could decrease their perception of the level of moral responsibility in comparison to a control group who received no particular information regarding addiction. Although we carried out the present data collection before Racine et al. (2017) published their study, our study is partly a conceptual replication of their text-only condition, for which they did not find a statistically significant effect on their free will responsibility scale.

Nahmias and Murray (2010) have noted that if one provides lay people with more concrete information about specific persons performing specific actions in specific circumstances, people engage their mind-reading abilities and consider the beliefs, desires, and intentions of agents, and thus more likely evoke judgments of free will and moral responsibility. Based on this idea, we exposed another group of participants to information depicting addiction as a brain disease and information about concrete actions needed to satisfy the addiction. We believed this focus on concrete behavior would invoke ideas about the agent’s intentions and therefore undo or counteract the potential impact of the neuromechanistic information.

The descriptors in Studies 1 and 2 that resulted in the lowest ratings of moral responsibility represented states and physical conditions of the individual, whereas the labels with the highest ascription of responsibility concerned behavior or capacities relating to behaviors (e.g., habit and willpower). One could argue that addiction as a state connotes elements of inaction, directing attention toward identity and a definition of someone as a certain kind of person. Having a status or identity does not necessarily mean that one acts out one’s identity. This point led us to investigate whether framing addiction as a state (being addicted) or as a behavior (performing actions to satisfy addiction) by changing the object of evaluation could influence judgments of moral responsibility. In summary, Study 3 tested two different ways of altering addiction conceptualizations: provision of information and framing addiction in terms of a behavior or a state.

#### Method

##### Sample and Design

Based on results from pilot data^2^, we chose the sample size such that it would give 80% power for a one-tailed test with a *p*- threshold of 0.05 for the comparison between the addiction state framing versus addictive behavior framing. Data from 1,062 Mturk participants were collected. The full design was a 2 (addiction type; within-person) by 3 (addiction information; between-person) by 2 (addiction framing; between-person) experimental design. Confidence intervals of mean differences were based on estimated marginal means from a repeated-measures ANOVA in SPSS 24.

#### Experimental Conditions and Measures

##### No Information

In the information control condition, participants did not get any information about addiction before they made responsibility judgments.

##### Brain Disease Information

In the brain disease information condition, participants received the following information, based on various internet resources:

In recent years, more and more research suggests that drug addiction can be viewed as a form of brain disease. The following text is based on information from the web page of the American Society of Addiction Medicine:

Research shows that the brain disease of addiction affects neurotransmission and interactions within the reward circuitry of the brain so that addictive behaviors substitute for normal healthy behaviors, and memories of previous experience with drugs trigger craving and desire for more addictive behavior. The disease creates distortions in thinking, feelings and perceptions. Addictive behaviors are manifestations of the brain disease, and the final result is a dysfunctional pursuit of rewards when seeking more drugs.

Here is another excerpt from a neuroscientist:

All drugs of abuse, from nicotine to heroin, provide a release of dopamine that creates a feeling of pleasure. In addition, this release of dopamine affects learning and memory. Addictive substances stimulate the same circuit in the brain that becomes activated by natural rewards such as sex and food. However, drugs overstimulate the circuit and the reward system responds with less production of dopamine—an adaptation similar to turning the volume down on a loudspeaker when noise becomes too loud. As a result of these adaptations, dopamine has less impact on the brain’s reward center so that the desired substance no longer gives as much pleasure as before. Addicts have to take more of the drug to obtain the same dopamine “high” because their brains have adapted—an effect known as tolerance. Now compulsion takes over, a reflection of how the normal machinery of motivation is no longer functioning.

##### Brain Disease + Agency Information

Participants in the brain disease + agency information condition first read the same information as in the Brain disease information condition; then, they received the following text:

An addiction to heroin typically requires planning and effort, for instance, planning how to obtain money, seeking a dealer, negotiating price, and preparing the drug before finally injecting or smoking it. An addiction to nicotine also requires planning and effort. Smokers addicted to nicotine have to buy cigarettes or tobacco, bring the cigarettes and perhaps a lighter or matches along when going out, find an appropriate place to smoke, and sometimes make plans about how to take smoking breaks that do not interfere with work or other activities.

##### Addiction States Versus Addictive Behavior

Orthogonal to the above three information conditions, approximately half of the respondents received the two questions “To what extent is a heroin user morally responsible for being addicted to heroin?” and “To what extent is a cigarette smoker morally responsible for being addicted to nicotine?”. This was the addiction as state condition. The other half received the two questions “To what extent is a heroin user morally responsible for actions performed to satisfy the addiction to heroin?” and “To what extent is a cigarette smoker morally responsible for actions performed to satisfy the addiction to nicotine?” This was the addiction as behavior condition. The responses were recorded on a scale from 0 (“not responsible at all”) to 5 (“fully responsible”).

#### Results and Discussion

Table 3 presents the mean levels of responsibility ratings for all conditions. An ANOVA suggested that the ratings varied between the information conditions (no information, brain, and rain + agency), *F*(2, 1,056) = 11.30, *p* < 0.0001, Partial Eta Squared = 0.02. The information describing addiction as a brain disease in a mechanistic and reductionistic language produced lower levels of moral responsibility than did the control condition, difference = −0.44, 95% CI [−0.26, −0.62]. When the brain disease description was followed by information about the plans and concrete actions addicted persons will have to make to satisfy their addiction, the moral responsibility ratings increased somewhat in comparison with the brain description only, difference = 0.19, 95% CI [0.00, 0.37]. However, these participants were still substantially more lenient than were those in the control group, difference = −0.25, 95% CI [−0.06, −0.44]. Thus, reminding people about the intentions, plans, and concrete actions involved in sustaining an addiction (i.e. *agency*) did not appear to cancel out the effect of conceptualizing addiction at the level of neural mechanisms.

**Table 3:** Ratings of moral responsibility for heroin and cigarette addiction in three information conditions by two framing conditions, Study 3.

	Addiction as state			Addiction as behavior		
		Heroin	Cigarettes		Heroin	Cigarettes
	*n*	*M* (SD)	*M* (SD)	*n*	*M* (SD)	*M* (SD)
No information	181	3.35 (1.35)	3.75 (1.26)	173	3.76 (1.29)	4.06 (1.21)
Brain	172	2.98 (1.29)	3.18 (1.29)	204	3.35 (1.26)	3.67 (1.11)
Brain + agency	180	3.21 (1.40)	3.43 (1.39)	152	3.54 (1.34)	3.75 (1.28)

When the object of evaluation was addictive behaviors, the ratings were 0.37 (one-sided 95% CI [0.25, inf.]) points higher than when the object of judgment was addictive states, *F* (1, 1,058) = 24.56, *p* < 0.0001, Partial Eta Squared = 0.02. Thus, lay people considered addicted persons to be more morally responsible for actions performed to satisfy an addiction than for the state of being addicted. This result is consistent with the idea that information about agents performing specific actions should evoke perceptions of free will and moral responsibility (Nahmias and Murray, 2010). Note that in principle, if people endorse a brain disease conceptualization and accept the mechanistic brain model of addiction, the responsibility for addictive states and addictive actions should be equally low.

Although the effect sizes were small, the data clearly showed that it is possible to manipulate people’s immediate judgments of responsibility for addictions. This suggests that those who provide information and have the power to frame questions about addiction, like the media and professionals in the justice and health care systems, also have the power to change people’s moral judgments about addicted individuals.

## General Discussion

In the present studies, we investigated the relation between conceptualizations of addiction and moral responsibility. To our knowledge, this is the first study where various labels and descriptions from the addiction literature are mapped onto a dimension of lay moral responsibility. Furthermore, the study showed that lay people’s moral judgments were malleable, which in past studies have been difficult to demonstrate.

Correlational data in Study 1 indicated that endorsement of labels that described addiction as a disease or disorder was associated with lower ratings of responsibility, whereas endorsement of labels relating to behavior and choice was associated with higher ratings of moral responsibility. This pattern was confirmed in Study 2 when lay people were asked to adopt certain perspectives and asked to make judgments about moral responsibility.

In Study 3, we observed that providing detailed information about brain mechanisms and neural changes following drug intake lowered ratings of moral responsibility. Adding information about the behaviors needed to satisfy an addiction reduced the effect of the brain mechanism information but did not fully cancel out the effect. A similar pattern of more responsibility for actions was also found when we manipulated the object of evaluation. Participants attributed more responsibility to addictive actions than addictive states.

In general, the studies demonstrate that conceptualizations of addictions can be consequential for judgments of moral responsibility. This may not seem to align with the findings of past research (Rather, 1991; Meurk et al., 2014; Racine et al., 2017). However, the study by Racine et al. (2017) showed similar tendencies as in our studies, and they noted that a more strongly worded message may be more successful in changing how people view addiction. Furthermore, past research has already documented that different conceptualizations, in the form of perceptions of different types of addictions, are consequential for moral judgments (Blomqvist, 2009; Rise et al., 2014). These past results on different types of addictions could be due to numerous factors, such as how common the addictive behavior is, how often people quit, how serious the health consequences are, what kind of people are associated with the behavior, and so on. In the present research, we either controlled for the average effect of the specific behavior (Study 1), or we manipulated conceptualizations while holding the behaviors constant (Studies 2 and 3). Thus, the present study shows how conceptualizations of addiction, irrespective of the nature of the specific addictive behavior, affect moral judgments.

The three studies used very different methods, from asking participants to rate how well a label represents addiction, to changing the object of evaluation. Still, we assume that the results reflect the same phenomenon, namely how the flexibility of people’s views of addiction can produce differences in their judgments of its moral consequences. People hold different views on different types of addictions, and this appears to be consequential for their moral judgments (Study 1). People are able to quickly change their inferences regarding moral consequences of addiction when we ask them to link addiction to other known concepts such as disease and habit (Study 2). People’s judgments of moral responsibility change when we provide new information or information that remind them of certain aspects of addiction, and people’s judgments change when we frame addiction as a behavior instead of a state (Study 3). Interestingly, regardless of the way addiction is malleable (i.e., addiction type, link to other concepts, provision of information, and framing of addiction), the relationship between the conceptualizations and their consequences for moral judgments appear to follow a predictable pattern, which is discussed below.

## Addiction models, attribution, and free will

Theoretically, the present results resonate well with the ideas described in the introductory sections. The explorative analyses of addiction labels in Studies 1 and 2 revealed that states and disease models of addiction were associated with lower levels of responsibility and labels implying reduced choice capacity or self-control failure were associated with higher levels of responsibility. Lay people’s intuitive judgments lie somewhere in the middle of the two extreme poles of moral responsibility, with only slight variation, depending on whether addiction is conceptualized as disease or choice/behavior. The placement of addiction in the middle of a moral responsibility continuum is consistent with recent models of addiction (Henden et al., 2013; Holton and Berridge, 2013; Heather, 2017a).

The results were also consistent with the idea that moral judgments are based on perceptions of controllability of cause (e.g., Weiner, 1995) or perceptions of intent (Shaver, 1985). Presumably, people think that concrete behaviors are controllable, whereas being addicted is not so controllable. This was particularly clear in Study 3, where we manipulated the object of evaluation, and observed higher ratings of responsibility for addictive behavior than for being addicted. Furthermore, the specific information about actions given after the neuromechanistic information also pointed to the potential of intentional control over addiction, and it appeared to reduce some of the effect of the neuromechanistic information.

In the introductory sections, we presented a psychological lay model of free will as degrees of agency. Addicts are agents who have capacities to decide and exercise control but who are also subject to internal and external constraints (cf. Nahmias, 2018). Most likely, lay people know that addicts have a strong desire for the drug, experience a lot of psychological distress and that they may not have many available alternative courses of actions. In other words, people may perceive addicts as having free will but not being fully free agents. Judged by the pattern of moral judgments in the present studies, the notion that addicts have free will and at the same time are unfree agents does not seem to represent a paradox for lay people. This also seemed to be the case in the study by Wiens and Walker (2015), where adopting a disease model did not have any impact on beliefs in free will but still reduced beliefs in agency.

Similarly, it appears that lay people see no contradiction in thinking of addicts as simultaneously *intentional* agents and unfree agents. Reminding lay people that consumption of a drug requires an elaborate series of planning, preparation, and effortful actions in advance of consumption in Study 3 (i.e., addicts are in effect agents with an intact intentional system) did not lead them to fully ignore the brain information. Perhaps the research participants were thinking that the elaborate efforts to satisfy the addiction could be propelled by a strong desire, thus bypassing the intentional system.

Even when asked to accept a mechanistic disease view, lay people were more willing to attribute moral responsibility for addictive actions than for states. This may reflect the perception that addicted persons have a choice when performing concrete actions but still have an underlying condition that limits agency and serves as an excuse for *being* addicted. This pattern of judgments suggests that lay people hold a model similar to the *disorder of choice model* advocated by Heather (2017b): “[…]what is needed is a model that continues to see addiction as behavior that people find extremely difficult to change while at the same time accepting the obvious fact of voluntary drug-seeking and – taking.” Although lay people’s perception of agency decreases when addiction is described as a disease, they still consider addicted individuals to be moral agents with a capacity for choice.

## Implications

This study among lay people provides evidence that conceptualizations of addiction matter for assignment of moral responsibility, with addictive labels related to choices and behaviors increasing the level of moral responsibility and labels related to brain disease lowering the level of responsibility. Based upon the present data it may, in principle, be possible to raise or lower the level of moral responsibility by manipulating the description of addiction. If one wishes a high level of moral responsibility for addiction, one could provide a minimal amount of information about the etiology and mechanisms of addiction and focus upon addictive actions. In contrast, if one wants a low level of moral responsibility, one could conceptualize addiction as a disease or disorder by providing information about brain mechanisms or using labels relating to disease and mental disorders. Motivational labels, like urge and desire, may be more neutral (Study 1) or imply an intermediate level of responsibility (Study 2).

The labels and descriptions used by, for example, the media and scientists might influence the public and policy makers, and, in turn, affect how addicted individuals are treated. Lay perceptions of moral responsibility of addictions have been shown to be a predictor of how much help addicted individuals deserve in the sense that higher levels of moral responsibility lowers the level of deservingness of help (e.g., Rise et al., 2014) and may thus function as a legitimation for policy decisions. We would be happy to see future research on consequences of moral responsibility of different conceptualizations of addiction for real life outcomes such as social interactions and policy decisions.

We do have to keep in mind that less responsibility is not necessarily beneficial for addicted individuals. Wiens and Walker (2015) found that people with a mild to moderate alcohol addiction experienced less control in relation to their drinking after being manipulated to adopt a disease model of addiction. Adopting a disease model did not reduce feelings of stigma more than adopting a psychosocial model. Similarly, it has been shown that lay models of psychiatric disorders based on biological mechanisms can increase pessimism about recovery and may increase the perception that people with psychological problems are dangerous (see Kvaale et al., 2013). Furthermore, a study by Kingree et al. (1999) suggested better outcomes in a 12-step program when participants felt more personally responsible for their addictions. On the other hand, one study showed that people who were informed that they had a genetic predisposition to alcoholism were more willing to sign up for a workshop on responsible drinking (Dar-Nimrod et al., 2013).

## Limitations, Strengths, and Conclusions

In cross-sectional studies, the relation between addiction conceptualizations and responsibility judgments can be confounded by variables relating to demographics and ideology (e.g., elder people could give higher endorsement due to familiarity with the concept and also be generally more punitive). This is not a problem in the present studies as we focused on within-person effects and used experimental manipulations. However, Study 1 did not give us any information about the direction of potential causal relations, and Study 2 only indicated consequences for responsibility judgments *given that* a certain conceptualization was accepted.

We used a direct measure of moral responsibility that is assumed to capture the process of assigning moral responsibility to events and behaviors (Weiner, 1995). Although it seems to be a common practice in experimental philosophy to use one-item measures for moral responsibility (see Cova and Kitano, 2014), this might be perceived as problematic in terms of measurement reliability. However, if we were to combine the two response measures (heroin and cigarette smoking) in Study 3 to an index, Cronbach’s alpha would be as high as 0.9.

The choice of ratings of moral responsibility as our only outcome measure limits our knowledge about specific real-life consequences of adopting different addiction models. Responsibility is a rather abstract concept believed to contribute to a range of outcomes (e.g., Weiner, 1995, 2006; Halkjelsvik and Rise, 2014).

The present studies showed that natural variations in conceptualizations of addiction may be consequential for judgments of moral responsibility, that different conceptualizations imply different moral judgments, and that conceptualizations are malleable through information and through changing the focus of evaluation (states versus actions). This means that the way people describe, teach about, and frame addiction could have implications for a range of behaviors that are based on moral judgments.

## Data Availability

The datasets generated for this study are available on request to the corresponding author.

## Ethics Statement

As the study involved anonymous data, voluntary participation, and did not ask for any information about participants’ health or any other sensitive data, the study did not require approval from an ethics committee according to Norwegian law. Participants were informed that their responses would be used in research and asked to consent by proceeding to the questionnaire.

## Author Contributions

The authors contributed equally to the article. JR drafted the introductory sections and the Discussion. TH performed statistical analyses and drafted the method and results sections. Both authors designed the studies, revised the manuscript, and approved the final version.

### Conflict of Interest Statement

The authors declare that the research was conducted in the absence of any commercial or financial relationships that could be construed as a potential conflict of interest.

## Footnotes

^1^Half the sample received a version of the questionnaire in which they were generally asked to think about what it means to be addicted; the other half of the sample was instructed to imagine that a person close to them was addicted to a given substance. This variable was only used as a covariate in the present study and had no substantial impact on responsibility judgments.

^2^The pilot data were from the same survey as the data reported in Study 2. The difference between a state vs. behavior condition in the pilot data was 0.2 points on a scale from 0 to 5, 95% CI [0.047–0.377], *F*(1, 43) = 6.70, *p* = 0.013. We later discovered that Albers and Lakens (2018) recommended *not* to calculate power directly based on pilot data effect sizes.
